# Electron Microburst Size Distribution Derived With AeroCube‐6

**DOI:** 10.1029/2019JA027651

**Published:** 2020-03-05

**Authors:** M. Shumko, A. T. Johnson, J. G. Sample, B. A. Griffith, D. L. Turner, T. P. O'Brien, O. Agapitov, J. B. Blake, S. G. Claudepierre

**Affiliations:** ^1^ Department of Physics Montana State University Bozeman MT USA; ^2^ Space Science Applications Laboratory The Aerospace Corportation El Segundo CA USA; ^3^ Space Sciences Laboratory University of California Berkeley CA USA; ^4^ Department of Atmospheric and Oceanic Sciences University of California Los Angeles CA USA

## Abstract

Microbursts are an impulsive increase of electrons from the radiation belts into the atmosphere and have been directly observed in low Earth orbit and the upper atmosphere. Prior work has estimated that microbursts are capable of rapidly depleting the radiation belt electrons on the order of a day; hence, their role to radiation belt electron losses must be considered. Losses due to microbursts are not well constrained, and more work is necessary to accurately quantify their contribution as a loss process. To address this question, we present a statistical study of >35 keV microburst sizes using the pair of AeroCube‐6 CubeSats. The microburst size distribution in low Earth orbit and the magnetic equator was derived using both spacecraft. In low Earth orbit, the majority of microbursts were observed, while the AeroCube‐6 separation was less than a few tens of kilometers, mostly in latitude. To account for the statistical effects of random microburst locations and sizes, Monte Carlo and analytic models were developed to test hypothesized microburst size distributions. A family of microburst size distributions were tested, and a Markov chain Monte Carlo sampler was used to estimate the optimal distribution of model parameters. Finally, a majority of observed microbursts map to sizes less than 200 km at the magnetic equator. Since microbursts are widely believed to be generated by scattering of radiation belt electrons by whistler mode waves, the observed microburst size distribution was compared to whistler mode chorus size distributions derived in prior literature.

## Key Points

- The dual AeroCube‐6 CubeSats simultaneously observed >35 keV microbursts at a variety of spatial separations ranging from 2 to 100 km
- In low Earth orbit the majority of microbursts have a size on the order of a few tens of km
- Mapped to the magnetic equator, the majority of microbursts are less than 200 km in size, corresponding to the size of chorus wave packets

## 1 Introduction

Since the discovery of the Van Allen radiation belts in the 1960s by Van Allen (1959) and Vernov and Chudakov (1960), decades of research have made headway in understanding the various particle acceleration and loss mechanisms. One of the extensively studied mechanisms responsible for particle acceleration and loss is wave‐particle scattering between whistler mode chorus waves and electrons (e.g., Abel & Thorne, 1998; Bortnik et al., 2008; Horne & Thorne, 2003; Meredith et al., 2002; Millan & Thorne, 2007; Thorne et al., 2005). Whistler mode chorus waves are typically generated by a temperature anisotropy of low‐energy electrons up to tens of kiloelectronvolts (keV) and are typically found in the ∼0–12 magnetic local times (MLTs) (Li et al., 2009; Li et al., 2009). Whistler mode chorus waves interact with radiation belt electrons and are widely believed to cause electron precipitation termed microbursts (e.g., Millan & Thorne, 2007).

Microbursts are a subsecond impulse of electrons that are observed by high‐altitude balloons and satellites in low Earth orbit (LEO) on radiation belt magnetic footprints ∼4–8 L‐shell (L) (e.g., Anderson & Milton, 1964; Breneman et al., 2017; Crew et al., 2016; Greeley et al., 2019; Lorentzen et al., 2001; Mozer et al., 2018; O'Brien et al., 2003; Tsurutani et al., 2013; Woodger et al., 2015), mostly in the dawn MLTs, and with an enhanced occurance rate during disturbed magnetospheric times (Douma et al., 2017; O'Brien et al., 2003). Microburst's role as a radiation belt electron loss mechanism has been estimated to be significant, with total radiation belt electron depletion due to microbursts estimated to be on the order of a day or less (Breneman et al., 2017; Douma et al., 2019; Lorentzen et al., 2001; O'Brien et al., 2004; Thorne et al., 2005). These average microburst loss estimates are not well constrained due to assumptions made regarding the microburst precipitation region.

One of the unconstrained microburst parameters that is critical to better quantify the role of microbursts as an instantaneous loss mechanism (the number of electrons lost per microburst) is their physical size. Historically, after the bremsstrahlung X‐ray signatures of microbursts were discovered by Anderson and Milton (1964), numerous studies investigated the size of predominately subhundred keV energy microbursts using other balloon flights in the mid‐1960s. Brown et al. (1965) used data from a pair of balloons separated by 150 km, mainly in longitude, and found that one third of all microbursts observed was temporally coincident. Trefall et al. (1966) then used the results from Brown et al. (1965) to model the probability that a microburst will be observed by two balloons as a function of the microburst radius, the radius of the precipitating area a balloon is sensitive to, and the balloon separation. Trefall et al. (1966) concluded that the microbursts reported by Brown et al. (1965) must have had a diameter of 230 km assuming a balloon has a circular field of view with a 140 km diameter (for electrons stopped at 100 km altitudes). Soon after, Barcus et al. (1966) used a pair of balloons and concluded that a microburst must have a <200 km longitudinal extent. Then Parks (1967) used data from a single balloon with four collimated scintillators oriented in different directions and found that the size of some mostly low energy microbursts to have a diameter of 80 ± 28 km, and others were less than 40 km.

More recently, direct observations of microburst electrons have been made by LEO spacecraft. Blake et al. (1996) found a microburst with a size of a few tens of kilometers using the the Solar Anomalous and Magnetospheric Particle Explorer (SAMPEX) and concluded that typically microbursts are less than a few tens of electron gyroradii in size (order of a few kilometers in LEO). Dietrich et al. (2010) used SAMPEX observations in another case study and concluded that the observed microbursts were smaller than 4 km. Crew et al. (2016) used the Focused Investigation of Relativistic Electron Bursts: Intensity, Range, and Dynamics (FIREBIRD‐II) CubeSats and found an example of a microburst larger than 11 km. Lastly, Shumko et al. (2018) also used FIREBIRD‐II to identify a microburst with a size greater than 51 ± 1 km. If anything, the large variation in prior results imply that there is a distribution of microburst scale sizes which this study aims to estimate.

Besides addressing the instantaneous radiation belt electron losses due to microbursts, the size distribution of individual microbursts is useful to identify the wave mode(s) responsible for scattering microbursts. By mapping the microburst size distribution in LEO to the magnetic equator, it can be compared to the wave sizes estimated in prior literature. This comparison can be used to identify the waves and their properties (e.g., amplitude or coherence) responsible for scattering microburst electrons.

This paper expands the prior microburst size case studies and addresses these two questions by analyzing microburst observations over a 3 year time period to estimate the microburst size distribution in LEO and the magnetic equator. The twin AeroCube‐6 (AC6) CubeSats are utilized for this study because they were ideally equipped to observe microbursts simultaneously over a span of 3 years, while their total separation varied between 2 and 800 km, mostly in latitude (in‐track in orbit). This paper first describes the AC‐6 mission, including their orbit and instrumentation in section 2. Section 3.1 develops the methodology used to identify microbursts observed by each spacecraft and how they were combined to make a list of simultaneously observed microbursts. Section 3.2 describes the methodology used to estimate the microburst size distributions in LEO and the magnetic equator as a function of AC6 separation. Then a model is developed in section 4 to shed light on how the compounding effects of a hypothesized microburst shape, random locations, and size distribution will be observed by AC6, a two‐point measurement platform. Various discrete and continuous microburst size distributions were tested, with a focus on discrete models due to their simple interpretation. Lastly, in section 5 we discuss these results and compare the microburst sizes estimated here to the size distribution of the whistler mode chorus waves that are believed to cause microbursts.

## 2 Instrumentation

**Figure 1:**
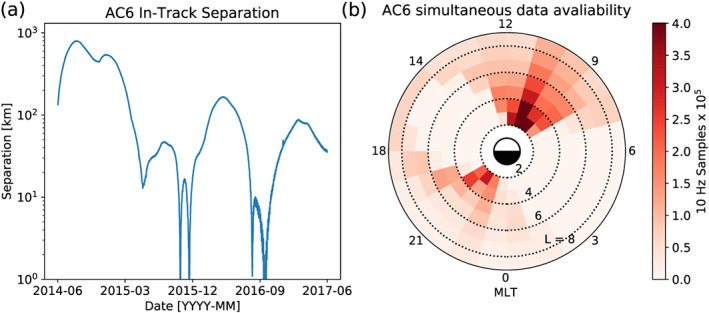
AC6 mission properties for (a) spacecraft separation and (b) number of simultaneous quality 10 Hz samples as a function of L and MLT.

The AC6 mission consists of a pair of 0.5U (10 × 10 × 5 cm) CubeSats built by The Aerospace Corporation and launched on 19 June 2014 into a 620 × 700 km, 98° inclination orbit. The two satellites, designated as AC6‐A and AC6‐B, separated after launch and drifted apart. Both AC6 units have an active attitude control system, which allows them to adjust the atmospheric drag experienced by each AC6 unit by orienting their solar panel “wings” with respect to the ram direction. By changing their orientation, the AC6 mission was able to achieve fine separation control and maintain a separation between 2 and 800 km, which was confirmed with GPS. Figure 1a shows the AC6 separation for the duration of the mission. Figure 1b shows where both AC6 units were taking 10 Hz data simultaneously as a function of L and MLT, which highlights that most data were taken at 8–12 MLT, an ideal local time for observing microbursts. Lastly, Figure 1b shows that over a 3 year period the AC6 orbit was roughly dawn‐dusk, Sun‐synchronous, and precessed 8 to 12 MLT in the dawn region. AC6's 8–12 MLT precession is ideal for sampling the region where microbursts are most likely to be observed (e.g., O'Brien et al., 2003) but the tradeoff is limited microburst size information in MLT.

Each AC6 unit is equipped with three Aerospace microdosimeters (licensed to Teledyne Microelectronics, Inc). The dosimeter used for this study, dos1, is identical on both AC6 units and has a 35 keV electron threshold. All AC6 dosimeters sample at 1 Hz in survey mode and 10 Hz in burst mode in the radiation belts (O'Brien et al., 2016). Since microburst duration is less than a second, only the 10 Hz data were used to identify microbursts.

## 3 Methodology

### 3.1 Microburst Detection

The first step to find microbursts observed simultaneously by AC6 is to identify them on each individual spacecraft. The detection method used to make the microburst data set is the burst parameter developed by O'Brien et al. (2003) and defined as $(n_{100}-a_{500})/\sqrt{1+a_{500}}$, where $n_{100}$ is the number of counts observed in 100 ms and $a_{500}$ is the 500 ms center running average. This definition is chosen because it balances the linear (n−a) and logarithmic (n/a) burstiness in a count time series. This algorithm has been successfully used in other microburst studies, mainly to study microbursts observed by SAMPEX (e.g., Blum et al., 2015; Douma et al., 2017, 2019; O'Brien et al., 2003). For AC6, a burst parameter threshold of $5\;{\text{counts}}^{1/2}$ was determined, by manual inspection of microbursts observed on an active day on 14 October 2016, to have a good trade‐off between false positive and false negative microburst detections.

With the microburst data sets from each AC6 unit in hand, data cleaning to remove microburst‐like transmitter noise were necessary. The transmitters on AC6 can cause unphysical count impulses in the dosimeters that resembles periodic trains of microbursts. One source of transmitter noise was observed when AC6 was in contact with the ground stations above the United States for data downloads and commanding; thus, the microburst detections made above the United States (that were mostly at low L) were discarded.

Another source of noise is crosslink transmissions between AC6‐A and AC6‐B. These transmissions occurred when either spacecraft transitioned from the survey mode to 10 Hz mode. This noise is sometimes not caught by the data quality flag, so the following empirically derived criteria were developed to remove those detections. The dosimeter with a 250 keV nominal electron threshold, dos2, was used because it had a nearly identical response to noise while rarely responded to microbursts. Since the transmitter noise is very periodic with a ≈0.2 s period, cross correlation (CC), and autocorrelation (AC) methods were applied to the dos1 and dos2 time series. Detections were discarded if the following two criteria were met: either dos1 or dos2 time series had a AC peak at a 0.2 or 0.4 s lag, and the dos1‐dos2 CC was greater than 0.9. The AC window was 2 s, and the dos1‐dos2 CC window was 1 s. The AC lag criteria alone sometimes falsely removed legitimate trains of microbursts, so the second criteria insured that the detection was removed if there was also an unphysically high correlation across an order of magnitude in energy.

**Figure 2:**
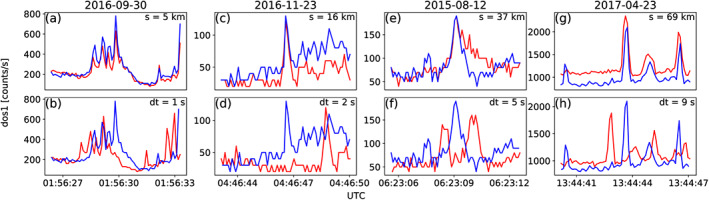
Examples of >35 keV microbursts observed simultaneously by AC6‐A in red and AC6‐B in blue. (a), (c), (e), and (g) show the temporally aligned time series when AC6 were separated by s= 5, 16, 37, and 69 km, respectively. The corresponding panels (b), (d), (f), and (h) show the spatially aligned time series which is made by shifting the AC6‐A time series in the above panels by the in‐track lag (annotated with dt) that would show any spatially correlated structures. The clear temporal correlation and lack of spatial correlation demonstrates that these events are microbursts.

Microbursts observed individually by AC6 were then merged into a data set of temporally correlated microbursts, that is, microbursts that were observed simultaneously by both AC6 units, with the following procedure. The general idea is that a microburst detected by one spacecraft will cross‐correlate well with the time series from the other spacecraft if it observed a similar microburst and poorly if there was no microburst observed by the other spacecraft. Thus, each microburst detection made by either spacecraft was cross‐correlated with the time series from the other spacecraft whether or not a microburst was observed by the other spacecraft. CC windows with 1 and 1.2 s widths, centered on the microburst peak, were used and three correlations calculated: two with the window start and end times aligned and one with the windows centered. The correlation assigned to each microburst was the maximum of the three. Microbursts detections that had a CC greater than 0.8 were considered temporally coincident. This CC threshold was chosen as it is low enough to accept user‐identified coincident microbursts superposed with noise and high enough to reject most noncoincident events. Figures 2a, 2c, 2e, and 2g show examples of microbursts observed by both AC6 units when they were separated by 5, 16, 37, and 69 km, respectively.

We also applied an additional criterion to eliminate stationary structures from the data set. These stationary structures are sometimes narrow in latitude, for example, curtains (Blake & O'Brien, 2016), and may be misidentified as microbursts. This criterion requires that the temporal CC must be greater than the spatial CC + 0.3. The spatial CC was calculated by shifting one spacecraft's time series by the in‐track lag to cross‐correlate at the same latitude. The 0.3 threshold was chosen so that the spatial correlation is much lower than the temporal correlation. Figures 2b, 2d, 2f, and 2h show the shifted time series to confirm that there were no spatially correlated, nonmicroburst structures present. Lastly, each event in the merged microburst data set was visually checked by two authors to remove poorly correlated events. After filtering out transmitter noise and applying the CC criteria, 662 simultaneous microburst detections were found and used in this study.

### 3.2 Microburst Size Distribution in LEO and Magnetic Equator

The temporally coincident microbursts, which from now on will be referred to as microbursts, were used to estimate the fraction of microbursts observed above AC6 separation, s. When AC6 observes a microburst at s, the microburst's size must be greater than s. This fact, along with the arguments presented in section 4 in Joy et al. (2002) who studied the most probable Jovian magnetopause and bow shock stand off distances, is used to investigate the dependence of the number of microbursts observed above s, as a function of s. This dependence is the microburst complementary cumulative distribution function $\bar{F}(s)$.

**Figure 3:**
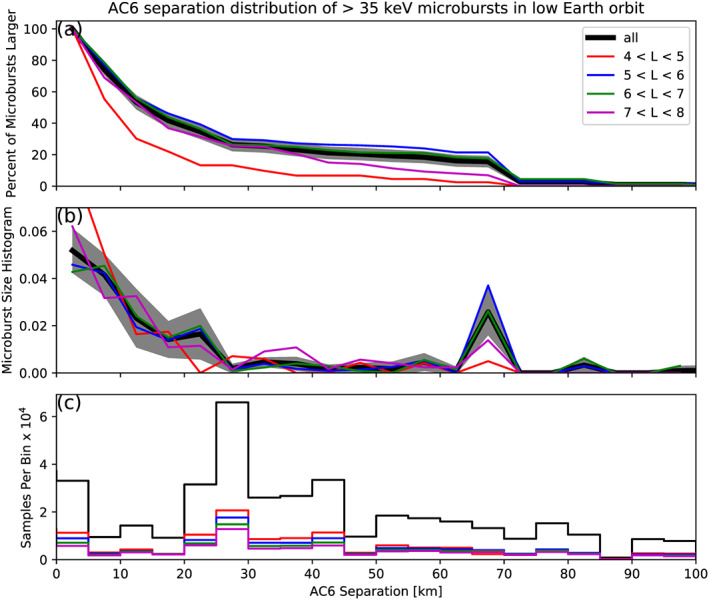
Microburst size distribution in low Earth orbit. Panel (a) shows the percent of microbursts observed above that separation after normalizing for the uneven AC6 sampling in separation. Panel (b) shows the microburst probability density (size histogram) as a function of separation. Lastly, panel (c) shows the normalization, that is, number of simultaneous samples AC6 observed as a function of separation. The colored lines show the distributions binned by L, and the thick black curve for the entire radiation belt ( 4<L<8). The gray shading around the black curve shows the 95% confidence interval uncertainty due to counting statistics, estimated and propagated from the unnormalized microburst detections. The uncertainty for the colored curves is larger since there are less events in those distributions, and are omitted for clarity.

The cumulative fraction of microbursts observed above s is the ratio of N(s), the normalized number of microbursts observed above s, to N(0), the normalized total number of microbursts observed

(1) $$\bar{F}(s)=\frac{N(s)}{N(0)}$$

where N(s) is defined by

(2) $$N(s)=\overset{\infty }{\underset{i=s}{\sum }}n_{i}\left(\frac{S_{max}}{S_{i}}\right)$$

where $n_{i}$ is the number of microbursts observed by AC6 in the ith separation bin. The normalization term $S_{max}/S_{i}$ is a ratio of the number of 10 Hz samples in the most sampled separation bin to the number of samples in the ith bin. This normalization factor corrects AC6's nonuniform sampling in separation; thus, $\bar{F}(s)$ can be interpreted as the fraction of microbursts observed above s assuming AC6 sampled evenly in separation. Microburst $\bar{F}(s)$ in LEO is shown by the black curve in Figure 3a for 4<L<8 and split into one L‐wide bins with the colored curves. The separation bin width used in Figure 3 is 5 km. To check for bias in $\bar{F}(s)$ due to the choice of separation bins, $\bar{F}(s)$ was resampled using other bin widths and offsets. Bin widths as large as 20–30 km and bin offsets did not qualitatively affect the curves in Figure 3a. The normalization, that is, the number of 10 Hz samples in each separation bin, is shown in Figure 3c.

The overall trend in Figure 3a shows a sudden cumulative probability drop‐off, followed by a shoulder up to s≈70 km where $\bar{F}(s)$ drops to nearly 0. The microburst size distribution does not have a clear dependence on L. An L shell trend was considered because it would reflect a similar trend in the scale size of the waves that generate microbursts. For example, Shen et al. (2019) found that chorus size is larger at larger L shell, but Agapitov et al. (2018) did not find such a trend. These two papers can not be directly compared, but their results show that there is no consensus on if the chorus wave size varies with L.

A large negative gradient of $\bar{F}(s)$ at some separation implies that microbursts must be smaller than that separation. To quantify this, Figure 3b shows the microburst probability density function (PDF), calculated by differentiating $\bar{F}(s)$. The microburst PDF shows a peak at s<30 km as well as a peak between 60 and 70 km separation. These PDF peaks are evidence of a sub‐30 km microburst population and larger microbursts observed up to 60–70 km separations. The shaded region around the black curves in Figures 3a and 3b shows the standard error due to counting statistics. The uncertainty due to false coincidence events, that is, two unrelated microbursts lining up in time by random chance, was also considered. The microburst duty cycle in a 1 min window ( ≈1L) around each microburst was calculated. The false coincidence probability is the square of the duty cycle and was found to be less than 5% for the majority of microbursts. The false coincidence probability for each microburst was then used to randomly remove microbursts and $\bar{F}(s)$ was recalculated in $10^{4}$ trials. The spread in the $\bar{F}(s)$ trial curves with microbursts randomly removed was much smaller than the uncertainty due to counting statistics alone.

The 60–70 km peak observed in Figure 3b is likely a combination of multiple factors that contribute to an imperfect normalization. Many of the microbursts in the 60–70 km bin were observed during a small number of radiation belt passes when the auroral electrojet index was consistently above 400 nT—at one time above 700 nT. Thus, it may have been an unusually active period when AC6 separation was approximately 65 km, which would bias the microburst distribution in a way that is difficult to correct with the observation time at that separation. Unfortunately, there are not enough microburst detections to investigate the dependence of the microburst distributions on geomagnetic activity.

**Figure 4:**
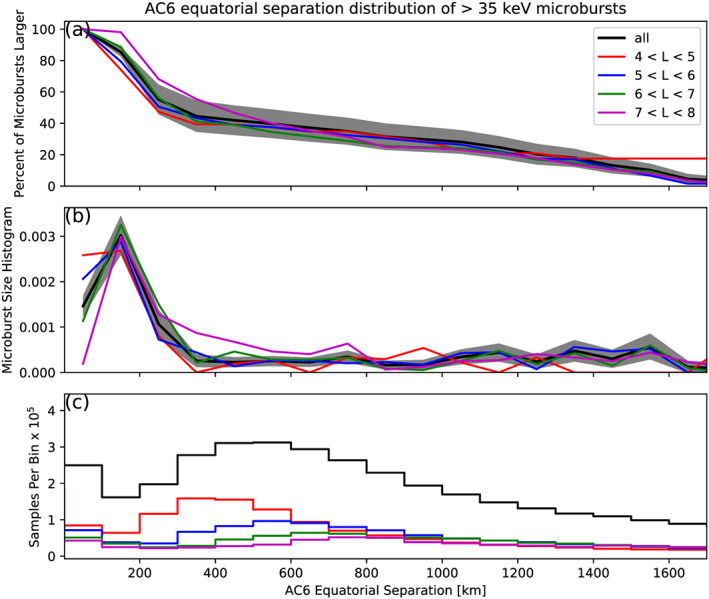
AC6 separation distribution of microburst sizes mapped to the magnetic equator in the same format as Figure 3.

To compare the microburst size to the size of their hypothesized progenitor waves, the spacecraft locations during observed microbursts were mapped to the magnetic equator using the Olson‐Pfitzer magnetic field model (Olson & Pfitzer, 1982), which is implemented with a Python wrapper for IRBEM‐Lib (Boscher et al., 2012). As previously stated, a microburst observed in LEO has a size larger than the spacecraft separation; hence, that microburst would also have a size larger than the spacecraft separation after it was mapped to the magnetic equator. Thus the procedure to estimate $\bar{F}(s)$ is identical to the LEO size distribution but with a different normalization. The normalization factors were calculated by mapping every quality AC6 sample to the magnetic equator and binning them by equatorial separation into 100 km wide bins. Most of the microbursts were observed when the AC6 equatorial separation was less than 200 km. Figure 4 shows the equatorial microburst size distribution in the same format as Figure 3. The equatorial and LEO $\bar{F}(s)$ trends, shown in Figures 3a and 4a, are qualitatively similar. Both initially decrease and then flatten out at separations greater than 20 km in LEO and approximately 200 km at the magnetic equator—corresponding well with the magnetic field scaling between LEO and the magnetic equator.

To identify the wave properties responsible for scattering microbursts, the spatial distributions of low ( <10 pT) and high ( >10 pT) amplitude lower band whistler model chorus waves were compared to the microburst distribution. A condensed data set from Agapitov et al. (2018) was used for this comparison, and the preliminary results are shown in Appendix A. A comprehensive comparison between wave and microburst distributions is beyond the scope of this work, but the preliminary results suggest that the equatorial microburst distribution more closely follows the >10 pT chorus distribution.

The results in Figures 3 and 4 show the fraction of microbursts observed above a spacecraft separation and do not fully represent the microbursts size distribution due to the compounding effects from the range of microburst sizes and random locations of microbursts near AC6. In other words, even if the microburst size is much larger than the AC6 separation, some fraction of those microbursts will be only observed by one AC6 spacecraft. Thus modeling is necessary to capture the compounding influence of these statistical effects on AC6.

## 4 Modeling the Distribution of Microburst Sizes

### 4.1 Monte Carlo and Analytic Models to Calculate $\bar{F}(s)$

To account for the effects due to microbursts randomly occurring around AC6 with an unknown distribution of microburst sizes, Monte Carlo and analytic models were developed. To estimate $\bar{F}(s)$, these models assume a hypothesized distribution of microburst sizes, expressed with a PDF p(d|θ), where θ are the dependent variables, and a microburst footprint shape. p(d|θ) can be understood as “the probability of observing a microburst of diameter d, given the parameters θ.” For simplicity, the microburst footprint is assumed to be circular with a diameter d. Various microburst size distributions were considered: a one‐size and two‐size microburst populations and continuous p(d|θ) such as Maxwell, Weibull, and log‐normal.

The Monte Carlo model first randomly scatters $10^{5}$ microburst centers in a 400 × 400 km grid around AC6. Then each microburst center was assigned a diameter, randomly picked from a p(d|θ) distribution after θ parameters were specified. Spacecraft A is placed at the origin, and Spacecraft B is placed along the positive y axis at various distances from Spacecraft A corresponding to the AC6 separation bins used in section 3.2. For each Spacecraft B location, the number of microbursts that encompass both spacecraft was counted. The modeled $\bar{F}(s)$ is the same as equation (1) without the normalization factor.

**Figure 5:**
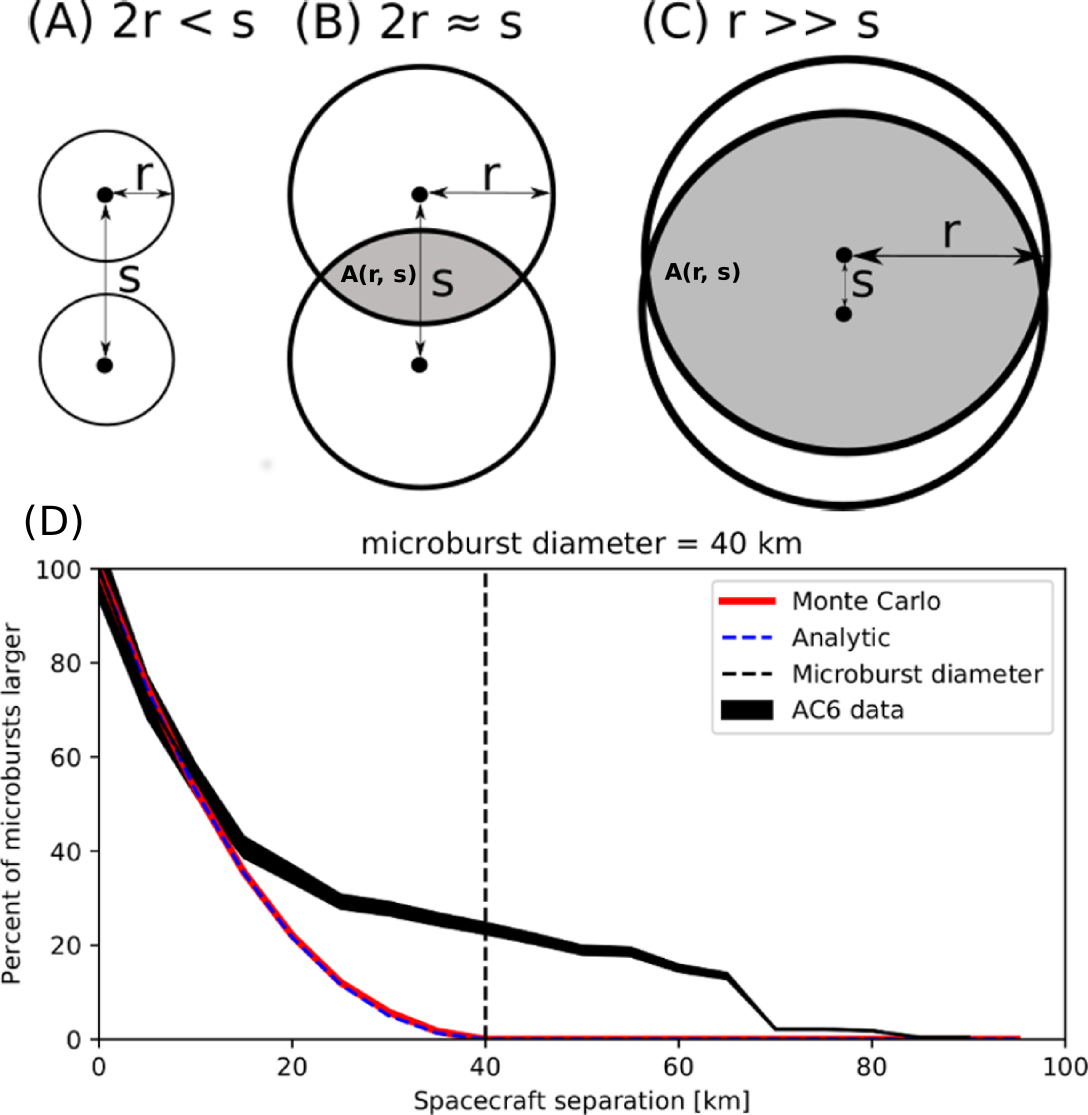
Panels (a)–(c) show the varying geometries of the analytic model. The two spacecraft are shown as black dots. The enclosing black circle around each spacecraft bounds the area where a microburst will be observed by at least one AC6 spacecraft if the microburst's center lies inside the circle. Panel (a) shows the case where microburst diameter is smaller than the AC6 separation, and all microbursts will be observed by either Unit A or B and never simultaneously. Panel (b) shows the intermediate case where the microburst diameter is comparable to the AC6 separation, and some fraction of microbursts, proportional to the circle intersection area A(r,s), will be observed simultaneously. Panel (c) shows the extreme case where nearly all microbursts will be observed by both spacecraft. Lastly, panel (d) shows $\bar{F}(s)$ from the AC6 data with a solid black line, and modeled MC and analytic $\bar{F}(s)$ curves for a single‐sized, d=40 km, microburst population.

The analytic model, while identical to the Monte Carlo model, highlights the geometrical concepts connecting p(d|θ) and $\bar{F}(s)$. For a microburst with d=2r≥s, there is an area between AC6 where that microburst will be observed by both spacecraft if the microburst's center lands there. Figures 5a–5c show this geometry with the two spacecraft indicated with black dots with varying relations between r and s. All microbursts whose center lies inside the circular area of radius r surrounding either spacecraft will be observed by that spacecraft. Figure 5a shows the geometry for microbursts smaller than the spacecraft separation where no microbursts are observed simultaneously. If it exists, the intersection of the two circular areas around both spacecraft defines another area, A(r,s) where a microburst will be observed by both spacecraft if the microburst center lands there. This area can be calculated using the circle‐circle intersection area equation,

(3) $$A(r,s)=2r^{2}\cos^{-1}\left(\frac{s}{2r}\right)-\frac{s}{2}\sqrt{4r^{2}-s^{2}}.$$

Example geometries where A(r,s)>0 are shown in Figures 5b and 5c. With this conceptual model and A(r,s), the analytic form of $\bar{F}(s)$ can be found and is derived in Appendix B. A concrete example is shown in Figure 5d. Figure 5d compares the observed, 4<L<8, $\bar{F}(s)$ curve to the Monte Carlo and analytic $\bar{F}(s)$ curves for a 40 km diameter microburst size population in red and dashed blue lines, respectively.

### 4.2 Methods for Estimating Optimal θ Parameters

At this stage we have all of the ingredients to model $\bar{F}(s)$ given a prescribed p(d|θ). For each p(d|θ) tested, the optimal θ parameters were estimated using traditional least squares regression and Bayesian inference. While we report the θ parameters that minimize least squares, this section focuses on Bayesian inference because it seamlessly incorporates statistical uncertainty in the data. The uncertainty in the data is passed on to uncertainty in θ, which is then no longer an optimal value, rather a distribution of values that is consistent with the observations and its uncertainty.

Bayesian inference is rooted in Bayes theorem of conditional probability. Given the observed $\bar{F}(s)$ as y, and model's dependent variables as θ, Bayes theorem can be written as

(4) $$p(\theta |y)=\frac{p(y|\theta )p(\theta )}{p(y)}.$$

p(θ) is the distribution of θ that describe our prior level of knowledge about each parameter; for example, from earlier microburst size studies, a microburst size must be less than 500 km in LEO. This is called the prior which is quantified by a PDF such as a normal or uniform distribution. Next term is the likelihood, p(y|θ), the conditional probability of obtaining y given a particular choice of θ. The likelihood probability is a probabilistic penalty function that quantifies the discrepancy between the modeled and observed $\bar{F}(s)$ in terms of the standard error. The resulting PDF of θ consistent with the observations is p(θ|y) known as the posterior distribution. The posterior is an update to our prior distributions, modified by the likelihood, that is, the data and its uncertainties. Here, the posterior is used to make inferences regarding the range of θ parameters that generate a $\bar{F}(s)$ that is consistent with the observations. The last parameter in Bayes theorem is p(y). p(y) is the marginal likelihood (also known as evidence) that describes the probability of obtaining y after marginalizing over the prior. Calculation of p(y) is difficult and often not necessary for model parameter estimation.

With all of the above terminology, the important takeaway is that the posterior distribution for each model parameter is interpreted as the range of our model's dependent parameters that are consistent with the observations. A 95% credible interval for each model parameter is reported here that is interpreted as follows: Assuming a hypothesized p(d|θ), there is a 95% probability that the true θ is bounded by the credible interval. To sample the posterior distribution, the θ parameter space is explored with a Markov chain Monte Carlo (MCMC) sampler. Briefly, a Markov Chain is a process where the state of a random variable depends only on the previous state. Hence, MCMC pseudorandomly samples the θ parameters based on the previous state of θ.

The first and one of the most popular MCMC is the Metropolis‐Hastings sampler (Hastings, 1970; Metropolis et al., 1953). While the Metropolis‐Hastings sampler is explained in detail in Metropolis et al. (1953) and Hastings (1970) and a good introduction given in Sambridge et al. (2006) as well as Sharma (2017), a brief overview is warranted. The Metropolis‐Hastings sampler samples the posterior distribution in N trials. Once an initial set of θ is randomly picked from the prior, the ith trial involves the following steps. First, calculate the posterior probability for $\theta_{i}$. Then pick a proposal $\theta_{i+1}$ to jump to, randomly picked near $\theta_{i}$ in parameter space. If the $\theta_{i+1}$ posterior probability is higher than $\theta_{i}$, the MCMC accepts the proposal and moves to $\theta_{i+1}$. If the posterior probability of $\theta_{i+1}$ is smaller than $\theta_{i}$, there is a random chance that $\theta_{i+1}$ will be accepted or rejected (if rejected, $\theta_{i+1}=\theta_{i}$ and a new proposal is generated). These accept/reject criteria allow the sampler to trend to more probable θ while also exploring the neighboring regions. After the N trials, a histogram is made using the accepted θs to produce the posterior distribution for each model parameter.

### 4.3 Estimating Optimal Parameters for LEO Microburst Size Models

**Figure 6:**
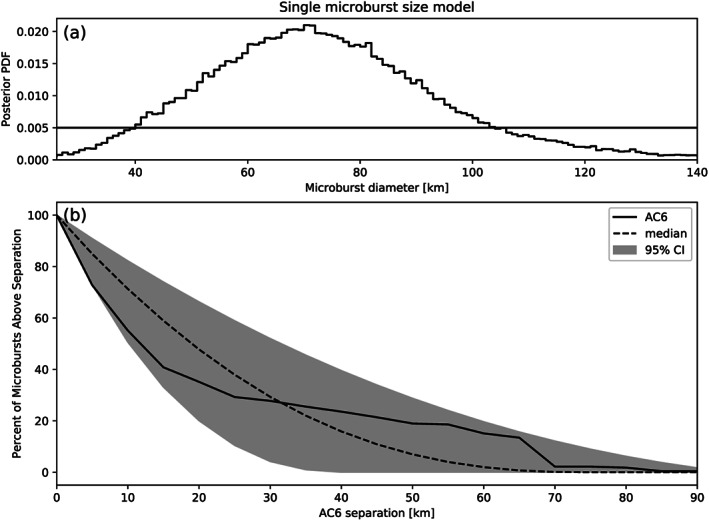
Range of plausible microburst sizes assuming all microbursts are one fixed size. Panel (a) shows the posterior probability density function of microburst diameters with the black curve. The posterior median microburst diameter is 73 km and the 95% credible interval is 38–129 km. A uniform prior between 0 and 200 km was assumed for this MCMC run and is shown with the horizontal black line. Panel (b) shows the $\bar{F}(s)$ curve from the AC6 data in black and the range of $\bar{F}(s)$ curves from the posterior. The median $\bar{F}(s)$ is shown with the dashed black curve and the gray shaded region corresponds to the 95% credible interval.

The MCMC sampler is first used to explore the simplest LEO microburst size model where all microbursts are one size. The microburst size PDF for this model can be expressed as

(5) $$p(d|d_{0})=\delta (d-d_{0})$$

where δ is the Dirac delta function and $d_{0}$ is the diameter of all microbursts according to this model. The posterior, that is, the range of d that are consistent with the observed $\bar{F}(s)$, is shown in Figure 6a. Assuming this model, there median microburst diameter is 73 km, and there is a 95% probability (credible interval) that the microburst diameter is between 38 and 129 km. The modeled median and the 95% credible interval $\bar{F}(s)$ curves are compared to the observations in Figure 6b. As a sanity check, the optimal size that minimizes least squares is 73 km. To quantitatively compare the median modeled and AC6 $\bar{F}(s)$ curves, the Kolmogorov‐Smirnov (K‐S) test was used. For this model the K‐S test statistic D=0.26 and the p value is p=0.53, so there is a 53% probability that the two $\bar{F}(s)$ curves were drawn from the same underlying distribution.

**Figure 7:**
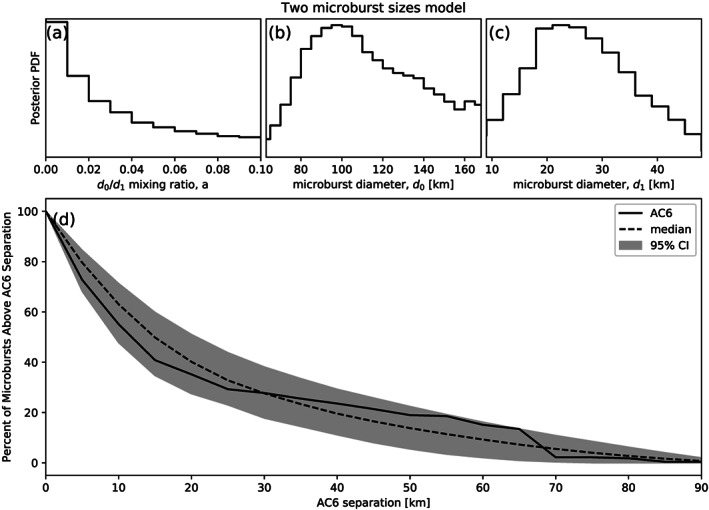
The range of plausible microburst sizes assuming the microburst size distribution is bimodal and consists of two sizes $d_{0}$ and $d_{1}$. The relative fraction of each size is a. Panel (a) shows the posterior distribution for a with has a median value of 0.02. The a prior was uniform between 0 and 0.2. Panel (b) shows the posterior distribution for $d_{0}$, the larger microburst population, estimated with a uniform prior between 50 and 200 km. The posterior median for $d_{0}$ is 122 km. Panel (c) shows the posterior distribution for $d_{1}$, the smaller microburst population, estimated using a uniform prior between 0 and 50 km with a posterior median diameter of 28 km. Panel (d) is similar to Figure 6b and shows the AC6 microburst $\bar{F}(s)$ with the solid black curve. To estimate the range of modeled $\bar{F}(s)$ curves in panel (d), a set of 1,000 random parameter triples ( a, $d_{0}$, and $d_{1}$) were drawn from the posterior and used to generate 1,000 $\bar{F}(s)$ curves. At each s the range of consistent $\bar{F}(s)$ were quantified by the median shown with the dashed black curve and 95% credible interval shown with the gray shading.

A slight generalization of the one‐size model is a two‐size microburst population model that assumes the following microburst PDF:

(6) $$p(d|d_{0},d_{1},a)=a\delta (d-d_{0})+(1-a)\delta (d-d_{1})$$

where the diameters of the two microburst populations are given by $d_{0}$ and $d_{1}$ and a is the parameter that quantifies the relative fractions of the two populations. The result of this model is shown in Figure 7, in a similar format as Figure 6. The fit is slightly better than the one‐size model, although that is to be expected given two more free model parameters. A majority, 98%, of microbursts have a diameter between 12 and 47 km with a rare population with a diameter between 76 and 234 km. The set of parameters that minimize least squares is 99.5 % of microbursts are small with a size of 21 km and the remaining 0.5% of microbursts have a 140 km size. For the two population model the K‐S test statistic is D=0.16 and p=0.98, which hints that the underlying microburst $\bar{F}(s)$ is bimodal.

Other, continuous PDFs were tested including Maxwellian (Maxwell‐Boltzmann), log‐normal, and Weibull. The range of model parameters that are consistent with the observed $\bar{F}(s)$ are presented in Appendix C. These distributions were chosen because they have the following realistic properties: They are continuous, can be symmetrical or asymmetrical, and approach 0 in the limit as r→0 (lower bound microburst size is ultimately limited by the electron gyroradius). Qualitatively, the two‐size model fits the observations the best out of all p(d) tested.

## 5 Discussion

The LEO microburst $\bar{F}(s)$ estimated in section 3.2 shows that a majority of coincident >35 keV microbursts were observed by AC6 when they were separated by less than a few tens of kilometers. The spatial distribution of predominately low‐energy microbursts determined here can be most directly compared with low‐energy microburst sizes determined from balloon observations. Our conclusion is most similar to Parks (1967) who reported that many >15 keV microbursts are less than 40 km in diameter, while others were on average 80 ± 28 km in diameter. The relatively small number of large >70 km microbursts observed by AC6 are consistent with the results from Brown et al. (1965) and Barcus et al. (1966), although the AC6 separation is mostly latitudinal, while Brown et al. (1965) and Barcus et al. (1966) used data from pairs of balloons that were separated predominantly in longitude.

Without knowledge of the microburst shape, a direct comparison between microburst observations made by AC6 and dual balloon observations is difficult. Trefall et al. (1966) discussed how a hypothetical circular microburst at the scattering location near the magnetic equator will be stretched into an ellipse with a semimajor axis in the longitudinal direction. This stretching effect should be explored further as it introduces an ambiguity from the eccentricity of the ellipse that prevents a direct latitudinal and longitudinal comparison.

When comparing our results to more recent spacecraft‐based studies, the AC6 distribution is similar to the >1 MeV microburst bouncing packet example shown in Blake et al. (1996) with a size of at least a few tens of kilometers. Furthermore, the AC6 microburst size distribution is larger than the sizes reported in Dietrich et al. (2010) who used very low frequency transmission paths and SAMPEX to conclude that >1 MeV microbursts must be smaller than 4 km from a small number of microbursts observed during one SAMPEX radiation belt pass. Dietrich et al. (2010) arrived at their conclusion by looking for temporal coincidence of microbursts and FAST events, subsecond very low frequency transmission perturbations, but the connection between FAST events and microbursts is not well understood. Lastly, our results are consistent with FIREBIRD‐II observations of 200 keV to >1 MeV microbursts. FIREBIRD‐II observed one microburst larger than 11 km (Crew et al., 2016), and a bouncing packet microburst that was larger than 51 km (Shumko et al., 2018).

The microburst PDF shown in Figure 3b suggests that the microburst size distribution is bimodal. This has been suggested before by Blake et al. (1996) who noted that the >150 keV and >1 MeV microbursts are not always well correlated, for example, Figure 10 in Blake et al. (1996). The quality of the AC6 data is insufficient to definitively conclude that there are two distinct microburst populations. The bimodal microburst population hypothesis can be better tested with an AC6‐like mission with energy resolution and homogeneous MLT coverage.

The model results from section 4 emphasize that care must be taken when comparing the $\bar{F}(s)$ curves observed by AC6 and the true microburst size distribution due to the compounding effect of an unknown microburst size distribution, unknown microburst shape, and random microburst locations near AC6. By assuming there is only one microburst size, the results in Figure 6 suggest that there is a 95% probability that the microburst diameter is somewhere between 38 and 129 km, a relatively wide range of values. On the other hand, the two‐size model has a smaller variance around the AC6 $\bar{F}(s)$, which is expected with the addition of two more free parameters. The two size model is interpreted as 98% of microbursts diameters are between 12 and 47 km and larger microbursts are uncommon.

A variety of continuous p(d|θ) such as the Maxwellian, Weibull, and log‐normal were also tested. While the continuous microburst PDFs are more realistic, there is no clear choice of which microburst PDF nature most closely resembles. The one‐ and two‐size models are simple to interpret, and the two‐size model qualitatively fits the observations the best out of all p(d) tested. Surely, nature does not only have two discrete microburst sizes. Rather, the current evidence and reasoning supports a bimodal and continuous PDF hypothesis. Due to lack of prior observations and theoretical predictions, it is difficult to identify and test a more appropriate p(d) hypothesis at this time.

The equatorial microburst $\bar{F}(s)$ estimated in section 3.2 and Figure 4b in particular shows that the majority of microbursts were observed when the equatorial AC6 separation was less than 200 km. We will now explore how these results compare to prior multipoint measurements of chorus source sizes made near the magnetic equator. The International Sun‐Earth Explorers 1 and 2 were used by Gurnett et al. (1979) to make one of the first direct chorus source scale measurements. Gurnett et al. (1979) estimated that the wave power correlation scale was on the order of a few hundred kilometers across the background magnetic field. Using the Cluster Wide Band Data measurements, Santolik et al. (2003) found the correlation scale of whistler mode chorus waves to be around 100 km near the source region at L≈4 and midnight MLT region. Furthermore, Turner et al. (2017) used the four Magnetospheric Multiscale Mission satellites and found that rising tone whistler mode chorus elements were phase coherent up to 70 km at L≈8. Agapitov et al. (2010, 2011, 2017, 2018) used multiple sets of spacecraft missions with wave measurements near the chorus source region to statistically show that the extent of chorus source region can extend from 600 km in the outer radiation belt to greater than 1,000 km in the outer magnetosphere. Most recently, Shen et al. (2019) used wave measurements from mostly the Van Allen Probes and found that the characteristic coherence size of lower band chorus waves transverse to the background magnetic field was ≈315 ± 32 km in the five to six L shell range. Qualitatively, the range of chorus sizes cited above is similar to our result—that most microburst observations map to less than 200 km at the magnetic equator.

More generally, small microburst sizes show that the waves responsible for scattering microburst electrons must have correlated properties on those scales. The wave properties necessary for scattering microburst electrons, for example, coherence, polarization, and wave normal angle can be identified by studying the waves properties that are only observed by multiple equatorial spacecraft at small separations. These properties can then aid wave‐particle scattering model development by constraining the wave properties and scattering modes. In turn, future models could then make predictions regarding the distribution of microburst sizes in LEO.

## 6 Conclusions

The twin AC6 CubeSats enabled the detailed statistical study of microburst sizes from a two point measurement platform. Roughly 60% of the >35 keV microbursts were simultaneously observed, while AC6 was separated by less than 20 km and the rest were observed up to ≈70 km separation. Modeling the microburst cumulative distribution function is essential to quantify the relationship between the number of microbursts observed as a function of separation to a hypothesized microburst size distributions. The AC6 microburst data, together with modeling, has hinted at the existence of a bimodal microburst size PDF with the majority of microbursts with a diameter smaller than 40 km and a rare microburst population with a diameter around 100 km. The bimodal size hypothesis may be more comprehensively addressed from LEO spacecraft with more simultaneous microburst observations, homogeneous MLT coverage, and differential energy channels. Moreover, to disentangle the compounding effect that affects two‐point microburst measurements, a X‐ray imager on a high‐altitude balloon can observe the atmospheric microburst footprint and determine the microburst size, shape, and any spatial correlations with little ambiguity.

When mapped to the magnetic equator, most microbursts were observed while the mapped AC6 separation was less than 200 km. This correlates well with the sizes of correlated high‐amplitude chorus waves, and it suggests that the wave properties crucial for scattering microbursts must be correlated over relatively small scales. By comprehensively studying the wave properties that are correlated on a few hundreds of kilometer scales, the dominant wave scattering modes may be identified.

## Figures and Tables

**Figure A1:**
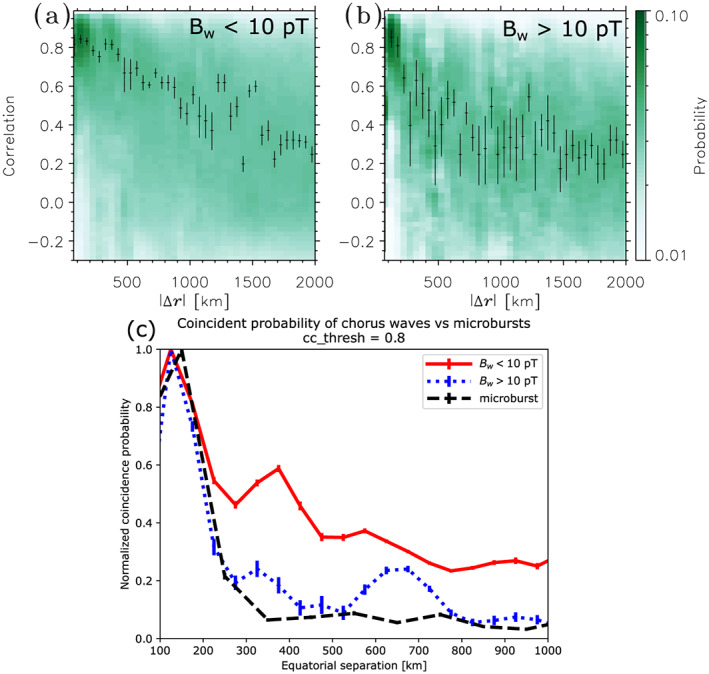
Comparison of the highly correlated lower band whistler mode chorus wave distribution estimated by Agapitov et al. (2018) to the AC6 equatorial microburst distribution. The chorus waves were split up by wave amplitude into a low ( $B_{w}<10$ pT) and high‐amplitude ( $B_{w}>10$ pT) subsets, and the distributions of chorus occurrence rates as a function of correlation and separation are shown in panels (a) and (b). The black dots in panels (a) and (b) show the mean correlation as a function of separation. Panel (c) compares the microburst and wave distributions. The red and blue curves show the probability of observing low‐ or high‐amplitude, highly correlated (correlation >0.8) chorus waves in each THEMIS separation bin. The black curve shows the AC6 equatorial microburst distribution in the same format. The errors bars in panel (c) show the standard error estimated using Poisson statistics.

## Appendix A Comparison of Microburst to Chorus Distributions

In this section we compare the equatorial distribution of microbursts sizes to the distribution of lower band whistler mode chorus waves near the magnetic equator. The wave data were obtained with the Time History of Events and Macroscale Interactions during Substorms (THEMIS) spacecraft from 2007 to 2017. Here we provide a brief overview of the procedure used to identify chorus waves which is described in more detail in Agapitov et al. (2018). The THEMIS search coil magnetometer instrument was used to make magnetic field measurements in six logarithmically spaced frequency channels between 1 and 4 kHz. These data were then used to cross‐correlate the chorus wave amplitudes between pairs of THEMIS spacecraft, and a data set of chorus waves was made.

This data set, shown in Agapitov et al. (2018), Figure 4—reproduced here in Figures A1a and A1b—was used in this preliminary study to estimate spatial distribution of chorus waves as a function of low and high wave amplitudes (10 pT threshold). In each 50 km THEMIS separation bin (perpendicular to the background magnetic field), the probability of observing a highly correlated chorus wave (CC greater than 0.8) was calculated. For each separation, this probability is defined as the number of correlated low‐ (high‐) amplitude waves, divided by the total number of low‐ (high‐) amplitude waves observed. The low‐ and high‐amplitude chorus wave distributions are shown in the red and blue curves in Figure A1c.

The AC6 equatorial microburst data set was analyzed in the same way to make a direct comparison. The probability of observing a coincident microburst in each equatorial separation bin (the cumulative estimates were not used) is shown with the black trace in Figure A1c.

Figure A1c shows a trend with a rapid probability drop‐off for >10 pT waves and microbursts within the first few hundred kilometers. The <10 pT wave probabilities also initially drop‐off and then remains relatively high at higher THEMIS separations. These results hint that the microburst probability distribution more closely tracks higher‐amplitude lower band whistler mode chorus wave distribution. A detailed comparison is outside the scope of this work, but a future study will need to address a few sources of systematic bias that may effect these results. A few biases include the magnetic field mapping error for the AC6 microbursts and much wider MLT coverage of THEMIS compared to AC6. With these biases in mind, other wave modes should also be compared.

## Appendix B Analytic Derivation of $\bar{F}(s)$

Here we derive the integral form of $\bar{F}(s)$ under the following assumptions:

1. microbursts are circular with radius r
2. microbursts are randomly and uniformly distributed around AC6.

Assuming the geometry in Figure 5 and the area A(r,s) given in equation( 3) (copied here for convenience),

(B1) $$A(r,s)=2r^{2}\cos^{-1}\left(\frac{s}{2r}\right)-\frac{s}{2}\sqrt{4r^{2}-s^{2}},$$

a circular microburst whose center lies in A(r,s) will be observed by both AC6 units and is counted in $\bar{F}(s)$. With A(r,s) we can derive the integral form of $\bar{F}(s)$ that accounts for the different spacecraft separations and microburst sizes that are distributed by a hypothesized p(r|θ).

First, we will account for the effects of various spacecraft separation, assuming that all microbursts are one size. As a reference, choose a radius, $r_{0},$ and spacecraft separation, $s_{0}$, such that $A(r_{0},s_{0})>0$. This condition implies that some number of microbursts, $n_{0}$, will be simultaneously observed. Now, if the spacecraft separation changes such that the area doubles, the second assumption implies that the number of microbursts observed during the same time interval must double as well. This can be expressed as

(B2) $$\frac{n_{0}}{A(r_{0},s_{0})}=\frac{n}{A(r,s)}$$

and interpreted as the conservation of the microburst area density. By rewriting equation( B2) as

(B3) $$n(r,s)=\left(\frac{n_{0}}{A(r_{0},s_{0})}\right)A(r,s)$$

it is more clear that the number of microbursts of size r observed at separation s is just A(r,s) scaled by a reference microburst area density. The cumulative number of microbursts observed above s is then

(B4) $$N(r,s)=\int_{s}^{\infty }n(r,s^{\prime })ds^{\prime }=\left(\frac{n_{0}}{A(r_{0},s_{0})}\right)\int_{s}^{\infty }A(r,s^{\prime })ds^{\prime }$$

and $\bar{F}(s)$ for a single r is

(B5) $$\bar{F}(s)=\frac{N(s)}{N(0)}=\frac{\int_{s}^{\infty }A(r,s^{\prime })ds^{\prime }}{\int_{0}^{\infty }A(r,s^{\prime })ds^{\prime }}$$

To derive the effects of a continuous microburst PDF on $\bar{F}(s)$, consider a microburst size distribution such as $p(r)=p_{1}\delta (r-r_{1})+p_{2}\delta (r-r_{2})+...$ The approach to estimate $\bar{F}(s)$ is similar, except now that we sum the weighted number of microbursts that each microburst size contributes to N(s); that is,

(B6) $$N(s)=\left(\frac{n_{0}}{A(r_{0},s_{0})}\right)\left(\int_{s}^{\infty }p_{1}A(r_{1},s^{\prime })ds^{\prime }+\int_{s}^{\infty }p_{2}A(r_{2},s^{\prime })ds^{\prime }+...\right)$$

where the $r_{1}$, $r_{2}$…terms in each integral came from integrating over the Dirac Delta function. The last step is to convert from the above sum into a continuous PDF:

(B7) $$N(s)=\left(\frac{n_{0}}{A(r_{0},s_{0})}\right)\overset{\infty }{\underset{s}{\int }}\overset{\infty }{\underset{0}{\int }}A(r,s^{\prime })p(r)drds^{\prime }.$$

With these considerations, $\bar{F}(s)$ is then given by

(B8) $$\bar{F}(s,\theta )=\frac{\overset{\infty }{\underset{s}{\int }}\overset{\infty }{\underset{0}{\int }}A(r,s^{\prime })p(r,\theta )drds^{\prime }}{\overset{\infty }{\underset{0}{\int }}\overset{\infty }{\underset{0}{\int }}A(r,s^{\prime })p(r,\theta )drds^{\prime }}.$$

## Appendix C Most Probable Parameter Values for Continuous Microburst PDFs

Besides, the one‐ and two‐size microburst models described in the main text, continuous PDFs such as the log‐normal, Weibull, and Maxwellian were fit and their optimal parameters presented here.

For the Maxwellian PDF, we assumed the following form

(C1) $$p(r|a)=\sqrt{\frac{2}{\pi }}\frac{r^{2}e^{-r^{2}/(2a^{2})}}{a^{3}}.$$

The range of a consistent with the observed data was found to be between 0 and 35 km. Next, the log‐normal distribution of the following form was used

(C2) $$p(r|\mu ,\sigma )=\frac{1}{\sigma r\sqrt{2\pi }}e^{\left(-{\left(ln(r)-ln(\mu )\right)}^{2}/(2\sigma^{2})\right)}$$

and the results are summarized in Table C1. Lastly, the offset Weibull distribution of the following form was tested:

(C3) $$p(r|c,r_{0},\lambda )=c{\left(\frac{r-r_{0}}{\lambda }\right)}^{c-1}exp\left(-{\left(\frac{r-r_{0}}{\lambda }\right)}^{c}\right).$$

for which the model parameters are summarized in Table C2.

**Table C1:** Range of Log‐Normal Model Parameters Consistent With the Observed AC6 $\bar{F}(s)$

Percentile (%)	μ	σ
2.5	1.8	0
50	21.8	0.4
97.5	52.0	1.1

**Table C2:** Range of Weibull Model Parameters Consistent With the Observed AC6 $\bar{F}(s)$

percentile (%)	c	$r_{0}$	λ
2.5	0.6	1.3	2.7
50	5.5	26.2	32
97.5	19.3	72.5	72.2

## References

[jgra55578-bib-0001] Abel, B. , & Thorne, R. M. (1998). Electron scattering loss in Earth's inner magnetosphere: 1. Dominant physical processes. Journal of Geophysical Research, 103(A2), 2385–2396.

[jgra55578-bib-0002] Agapitov, O. , Blum, L. W. , Mozer, F. S. , Bonnell, J. W. , & Wygant, J. (2017). Chorus whistler wave source scales as determined from multipoint Van Allen Probe measurements. Geophysical Research Letters, 44, 2634–2642. 10.1002/2017GL072701

[jgra55578-bib-0003] Agapitov, O. , Krasnoselskikh, V. , Dudok de Wit, T. , Khotyaintsev, Y. , Pickett, J. S. , Santolik, O. , & Rolland, G. (2011). Multispacecraft observations of chorus emissions as a tool for the plasma density fluctuations' remote sensing. Journal of Geophysical Research, 116, A09222 10.1029/2011JA016540

[jgra55578-bib-0004] Agapitov, O. , Krasnoselskikh, V. , Zaliznyak, Y. , Angelopoulos, V. , Le Contel, O. , & Rolland, G. (2010). Chorus source region localization in the Earth's outer magnetosphere using THEMIS measurements. Annales Geophysicae, 28(6), 1377–1386. 10.5194/angeo-28-1377-2010

[jgra55578-bib-0005] Agapitov, O. , Mourenas, D. , Artemyev, A. , Mozer, F. , Bonnell, J. , Angelopoulos, V. , & Krasnoselskikh, V. (2018). Spatial extent and temporal correlation of chorus and hiss: Statistical results from multipoint THEMIS observations. Journal of Geophysical Research: Space Physics, 123, 8317–8330. 10.1029/2018JA025725

[jgra55578-bib-0006] Anderson, K. A. , & Milton, D. W. (1964). Balloon observations of X rays in the auroral zone: 3. High time resolution studies. Journal of Geophysical Research, 69(21), 4457–4479. 10.1029/JZ069i021p04457

[jgra55578-bib-0007] Barcus, J. , Brown, R. , & Rosenberg, T. (1966). Spatial and temporal character of fast variations in auroral‐zone X rays. Journal of Geophysical Research, 71(1), 125–141.

[jgra55578-bib-0008] Blake, J. B. , Looper, M. D. , Baker, D. N. , Nakamura, R. , Klecker, B. , & Hovestadt, D. (1996). New high temporal and spatial resolution measurements by SAMPEX of the precipitation of relativistic electrons. Advances in Space Research, 18(8), 171–186. 10.1016/0273-1177(95)00969-8 11538959

[jgra55578-bib-0009] Blake, J. B. , & O'Brien, T. P. (2016). Observations of small‐scale latitudinal structure in energetic electron precipitation. Journal of Geophysical Research: Space Physics, 121, 3031–3035. 10.1002/2015JA021815

[jgra55578-bib-0010] Blum, L. , Li, X. , & Denton, M. (2015). Rapid MeV electron precipitation as observed by SAMPEX/HILT during high‐speed stream‐driven storms. Journal of Geophysical Research: Space Physics, 120, 3783–3794. 10.1002/2014JA020633

[jgra55578-bib-0011] Bortnik, J. , Thorne, R. , & Inan, U. S. (2008). Nonlinear interaction of energetic electrons with large amplitude chorus. Geophysical Research Letters, 35, L21102 10.1029/2008GL035500

[jgra55578-bib-0012] Boscher, D. , Bourdarie, S. , O'Brien, P. , Guild, T. , & Shumko, M. (2012). Irbem‐lib library.

[jgra55578-bib-0013] Breneman, A. , Crew, A. , Sample, J. , Klumpar, D. , Johnson, A. , & Agapitov, O. (2017). Observations directly linking relativistic electron microbursts to whistler mode chorus: Van Allen Probes and FIREBIRD II. Geophysical Research Letters, 44, 11,265–11,272. 10.1002/2017GL075001

[jgra55578-bib-0014] Brown, R. , Barcus, J. , & Parsons, N. (1965). Balloon observations of auroral zone X rays in conjugate regions. 2. microbursts and pulsations. Journal of Geophysical Research, 70, 2599–2612. 10.1029/JZ070i011p02599

[jgra55578-bib-0015] Crew, A. B. , Spence, H. E. , Blake, J. B. , Klumpar, D. M. , Larsen, B. A. , O'Brien, T. P. , & Widholm, M. (2016). First multipoint in situ observations of electron microbursts: Initial results from the NSF FIREBIRD II mission. Journal of Geophysical Research: Space Physics, 121, 5272–5283. 10.1002/2016JA022485

[jgra55578-bib-0016] Dietrich, S. , Rodger, C. J. , Clilverd, M. A. , Bortnik, J. , & Raita, T. (2010). Relativistic microburst storm characteristics: Combined satellite and ground‐based observations. Journal of Geophysical Research, 115, A12240 10.1029/2010JA015777

[jgra55578-bib-0017] Douma, E. , Rodger, C. J. , Blum, L. W. , & Clilverd, M. A. (2017). Occurrence characteristics of relativistic electron microbursts from SAMPEX observations. Journal of Geophysical Research: Space Physics, 122, 8096–8107. 10.1002/2017JA024067

[jgra55578-bib-0018] Douma, E. , Rodger, C. , Blum, L. , O'Brien, T. , Clilverd, M. , & Blake, J. (2019). Characteristics of relativistic microburst intensity from SAMPEX observations. Journal of Geophysical Research: Space Physics, 124, 5627–5640. 10.1029/2019JA026757

[jgra55578-bib-0019] Greeley, A. , Kanekal, S. , Baker, D. , Klecker, B. , & Schiller, Q. (2019). Quantifying the contribution of microbursts to global electron loss in the radiation belts. Journal of Geophysical Research: Space Physics, 124, 1111–1124. 10.1029/2018JA026368

[jgra55578-bib-0020] Gurnett, D. , Anderson, R. , Scarf, F. , Fredricks, R. , & Smith, E. (1979). Initial results from the ISEE‐1 and‐2 plasma wave investigation. Space Science Reviews, 23(1), 103–122.

[jgra55578-bib-0021] Hastings, W. K. (1970). Monte Carlo sampling methods using Markov chains and their applications.

[jgra55578-bib-0022] Horne, R. B. , & Thorne, R. M. (2003). Relativistic electron acceleration and precipitation during resonant interactions with whistler‐mode chorus. Geophysical Research Letters, 30(10), 1527 10.1029/2003GL016973

[jgra55578-bib-0023] Joy, S. , Kivelson, M. , Walker, R. , Khurana, K. , Russell, C. , & Ogino, T. (2002). Probabilistic models of the Jovian magnetopause and bow shock locations. Journal of Geophysical Research, 107(A10), SMP–17. 10.1029/2001JA009146

[jgra55578-bib-0024] Li, W. , Thorne, R. , Angelopoulos, V. , Bonnell, J. , McFadden, J. , Carlson, C. , & Auster, H. (2009). Evaluation of whistler‐mode chorus intensification on the nightside during an injection event observed on the THEMIS spacecraft. Journal of Geophysical Research, 114, A00C14 10.1029/2008JA013554

[jgra55578-bib-0025] Li, W. , Thorne, R. M. , Angelopoulos, V. , Bortnik, J. , Cully, C. M. , Ni, B. , & Magnes, W. (2009). Global distribution of whistler‐mode chorus waves observed on the THEMIS spacecraft. Geophysical Research Letters, 36, L09104 10.1029/2009GL037595

[jgra55578-bib-0026] Lorentzen, K. R. , Blake, J. B. , Inan, U. S. , & Bortnik, J. (2001). Observations of relativistic electron microbursts in association with VLF chorus. Journal of Geophysical Research, 106(A4), 6017–6027. 10.1029/2000JA003018

[jgra55578-bib-0027] Lorentzen, K. R. , Looper, M. D. , & Blake, J. B. (2001). Relativistic electron microbursts during the GEM storms. Geophysical Research Letters, 28(13), 2573–2576. 10.1029/2001GL012926

[jgra55578-bib-0028] Meredith, N. , Horne, R. , Summers, D. , Thorne, R. , Iles, R. , Heynderickx, D. , & Anderson, R. (2002). Evidence for acceleration of outer zone electrons to relativistic energies by whistler mode chorus. Annales Geophysicae, 20, 967–979.

[jgra55578-bib-0029] Metropolis, N. , Rosenbluth, A. W. , Rosenbluth, M. N. , Teller, A. H. , & Teller, E. (1953). Equation of state calculations by fast computing machines. The Journal of Chemical Physics, 21(6), 1087–1092. 10.1063/5.0309018 41342507

[jgra55578-bib-0030] Millan, R. , & Thorne, R. (2007). Review of radiation belt relativistic electron losses. Journal of Atmospheric and Solar‐Terrestrial Physics, 69(3), 362–377. 10.1016/j.jastp.2006.06.019

[jgra55578-bib-0031] Mozer, F. S. , Agapitov, O. V. , Blake, J. B. , & Vasko, I. Y. (2018). Simultaneous observations of lower band chorus emissions at the equator and microburst precipitating electrons in the ionosphere. Geophysical Research Letters, 45, 511–516. 10.1002/2017GL076120

[jgra55578-bib-0032] O'Brien, T. P. , Blake, J. B. , & W., G. J. (2016). AeroCube‐6 Dosimeter Data README (Tech. Rep. No. TOR‐2016‐01155): The Aerospace Corporation.

[jgra55578-bib-0033] O'Brien, T. P. , Looper, M. D. , & Blake, J. B. (2004). Quantification of relativistic electron microburst losses during the GEM storms. Geophysical Research Letters, 31, L04802 10.1029/2003GL018621

[jgra55578-bib-0034] O'Brien, T. P. , Lorentzen, K. R. , Mann, I. R. , Meredith, N. P. , Blake, J. B. , Fennell, J. F. , & Anderson, R. R. (2003). Energization of relativistic electrons in the presence of ULF power and MeV microbursts: Evidence for dual ULF and VLF acceleration. Journal of Geophysical Research: Space Physics, 108(A8), 1329 10.1029/2002JA009784

[jgra55578-bib-0035] Olson, W. P. , & Pfitzer, K. A. (1982). A dynamic model of the magnetospheric magnetic and electric fields for July 29, 1977. Journal of Geophysical Research, 87(A8), 5943–5948. 10.1029/JA087iA08p05943

[jgra55578-bib-0036] Parks, G. K. (1967). Spatial characteristics of auroral‐zone X‐ray microbursts. Journal of Geophysical Research, 72(1), 215–226.

[jgra55578-bib-0037] Sambridge, M. , Gallagher, K. , Jackson, A. , & Rickwood, P. (2006). Trans‐dimensional inverse problems, model comparison and the evidence. Geophysical Journal International, 167(2), 528–542.

[jgra55578-bib-0038] Santolik, O. , Gurnett, D. , Pickett, J. , Parrot, M. , & Cornilleau‐Wehrlin, N. (2003). Spatio‐temporal structure of storm‐time chorus. Journal of Geophysical Research, 108(A7), 1278 10.1029/2002JA009791

[jgra55578-bib-0039] Sharma, S. (2017). Markov chain Monte Carlo methods for Bayesian data analysis in astronomy. Annual Review of Astronomy and Astrophysics, 55, 213–259.

[jgra55578-bib-0040] Shen, X. C. , Li, W. , Ma, Q. , Agapitov, O. , & Nishimura, Y. (2019). Statistical analysis of transverse size of lower band chorus waves using simultaneous multisatellite observations. Geophysical Research Letters, 46, 5725–5734. 10.1029/2019GL083118

[jgra55578-bib-0041] Shumko, M. , Sample, J. , Johnson, A. , Blake, B. , Crew, A. , Spence, H. , & Handley, M. (2018). Microburst scale size derived from multiple bounces of a microburst simultaneously observed with the FIREBIRD‐II CubeSats. Geophysical Research Letters, 45, 8811–8818. 10.1029/2018GL078925

[jgra55578-bib-0042] Thorne, R. M. , O'Brien, T. P. , Shprits, Y. Y. , Summers, D. , & Horne, R. B. (2005). Timescale for MeV electron microburst loss during geomagnetic storms. Journal of Geophysical Research, 110, A09202 10.1029/2004JA010882

[jgra55578-bib-0043] Trefall, H. , Bjordal, J. , Ullaland, S. , & Stadsnes, J. (1966). On the extension of auroral‐zone X‐ray microbursts. Journal of Atmospheric and Terrestrial Physics, 28(2), 225–233.

[jgra55578-bib-0044] Tsurutani, B. T. , Lakhina, G. S. , & Verkhoglyadova, O. P. (2013). Energetic electron ( >10 keV) microburst precipitation, ∼ 5–15 s X‐ray pulsations, chorus, and wave‐particle interactions: A review. Journal of Geophysical Research: Space Physics, 118, 2296–2312. 10.1002/jgra.50264

[jgra55578-bib-0045] Turner, D. , Lee, J. , Claudepierre, S. , Fennell, J. , Blake, J. , Jaynes, A. , Leonard, T. , Wilder, F. D. , Ergun, R. E. , Baker, D. N. , Cohen, I. J. , Mauk, B. H. , Strangeway, R. J. , Hartley, D. P. , Kletzing, C. A. , Breuillard, H. , Le Contel, O. , Khotyaintsev, Y. V. , Torbert, R. B. , Allen, R. C. , Burch, J. L. , & Santolik, O. (2017). Examining coherency scales, substructure, and propagation of whistler mode chorus elements with Magnetospheric Multiscale (MMS). Journal of Geophysical Research: Space Physics, 122, 11,201–11,226. 10.1002/2017JA024474

[jgra55578-bib-0046] Van Allen, J. A. (1959). The geomagnetically trapped corpuscular radiation. Journal of Geophysical Research, 64(11), 1683–1689. 10.1029/JZ064i011p01683

[jgra55578-bib-0047] Vernov, S. , & Chudakov, A. (1960). Investigation of radiation in outer space. International Cosmic Ray Conference, 3, 19.

[jgra55578-bib-0048] Woodger, L. , Halford, A. , Millan, R. , McCarthy, M. , Smith, D. , Bowers, G. , & Liang, X. (2015). A summary of the BARREL campaigns: Technique for studying electron precipitation. Journal of Geophysical Research: Space Physics, 120, 4922–4935. 10.1002/2014JA020874 26937330 PMC4758627

